# 2-(Anthracen-9-yl)-10-meth­oxy­benzo[*h*]quinoline acetone hemisolvate

**DOI:** 10.1107/S1600536812031807

**Published:** 2012-07-25

**Authors:** Zhenming Dong, Bo Liu, Xinxin Cui, Yufang Liu

**Affiliations:** a School of Chemistry and Chemical Engineering, Shanxi University, Taiyuan 030006, People’s Republic of China; b Institute of Chemistry, School of Science, Beijing Jiaotong University, Beijing 100044, People’s Republic of China

## Abstract

The asymmetric unit of the title structure, C_28_H_19_NO·0.5C_3_H_6_O, comprises one 2-(anthracen-9-yl)-10-meth­oxy­benzo[*h*]­quinoline mol­ecule and an acteone mol­ecule with an occupany of 0.5. The solvent mol­ecule is disordered around a centre of symmetry. Its occupancy was determined from NMR data and kept fixed during the refinement. The two conjugated ring systems of the mol­ecule are almost perpendicular to each other; the inter­planar angle between the anthracene and quinoline ring systems is 84.9 (2)°.

## Related literature

For the structure and synthesys of a related compound, see: Dong *et al.* (2011 ▶). For background information on quinoline derivatives, see: Kouznetsov *et al.* (2005 ▶); Maguire *et al.* (1994 ▶).
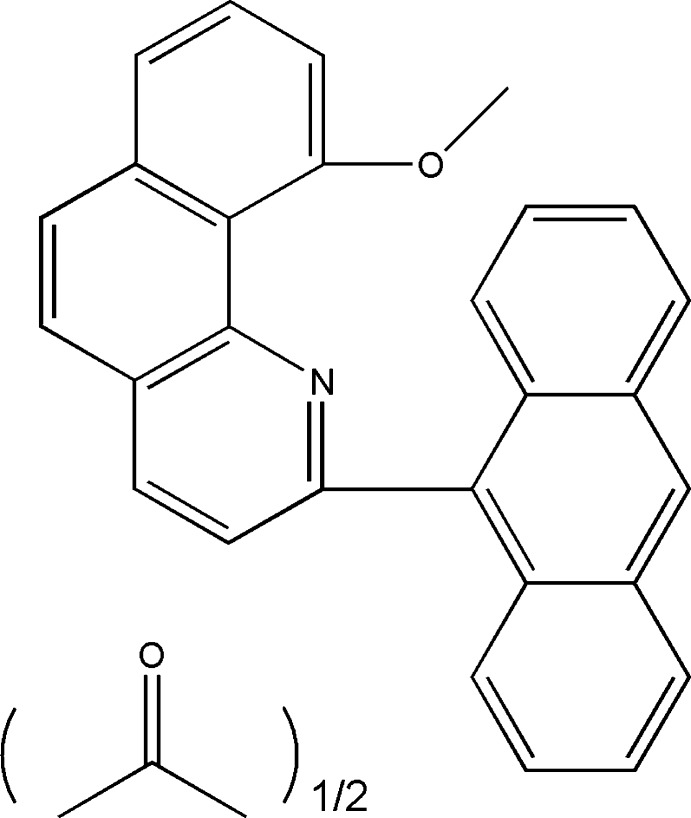


## Experimental

### 

#### Crystal data

C_28_H_19_NO·0.5C_3_H_6_O
*M* *_r_* = 414.48
Triclinic, 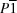

*a* = 9.198 (3) Å
*b* = 10.690 (4) Å
*c* = 11.130 (4) Å
α = 95.224 (4)°
β = 91.484 (5)°
γ = 94.064 (5)°
*V* = 1086.6 (6) Å^3^
*Z* = 2
Mo *K*α radiation
μ = 0.08 mm^−1^
*T* = 293 K
0.30 × 0.20 × 0.20 mm

#### Data collection

Bruker SMART APEX CCD diffractometer
Absorption correction: multi-scan (*SADABS*; Sheldrick, 1996 ▶) *T*_min_ = 0.977, *T*_max_ = 0.985
4167 measured reflections
3688 independent reflections
2474 reflections with *I* > 2σ(*I*)
*R*_int_ = 0.011

#### Refinement

*R*[*F* ^2^ > 2σ(*F* ^2^)] = 0.069
*wR*(*F* ^2^) = 0.160
*S* = 1.01
3688 reflections
300 parameters
24 restraints
H-atom parameters constrained
Δρ_max_ = 0.58 e Å^−3^
Δρ_min_ = −0.36 e Å^−3^

### 

Data collection: *SMART* (Bruker, 2001 ▶); cell refinement: *SAINT* (Bruker, 2001 ▶); data reduction: *SAINT*; program(s) used to solve structure: *SHELXS97* (Sheldrick, 2008 ▶); program(s) used to refine structure: *SHELXL97* (Sheldrick, 2008 ▶); molecular graphics: *SHELXTL* (Sheldrick, 2008 ▶); software used to prepare material for publication: *SHELXTL*.

## Supplementary Material

Crystal structure: contains datablock(s) I, global. DOI: 10.1107/S1600536812031807/fy2041sup1.cif

Structure factors: contains datablock(s) I. DOI: 10.1107/S1600536812031807/fy2041Isup2.hkl

Supplementary material file. DOI: 10.1107/S1600536812031807/fy2041Isup3.cml

Additional supplementary materials:  crystallographic information; 3D view; checkCIF report

## Figures and Tables

**Fig. 1.**
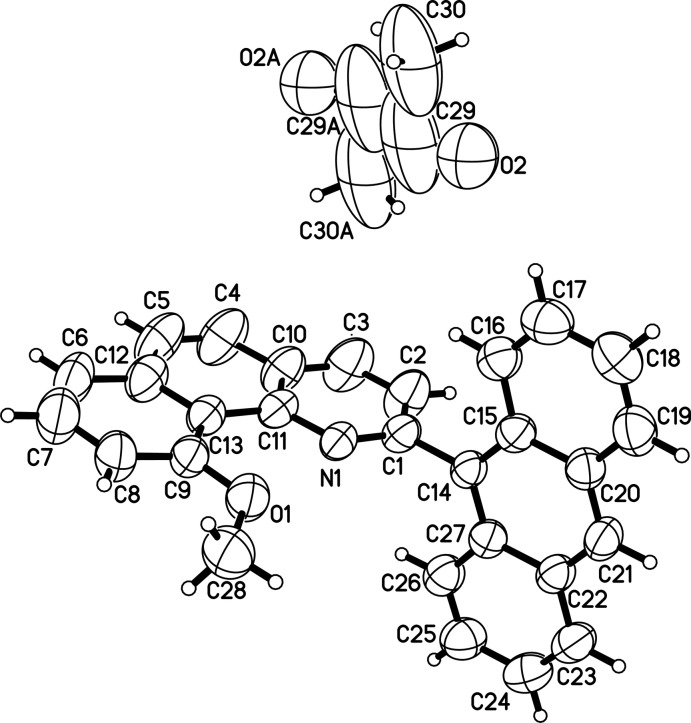
The molecular structure of the title compound with displacement ellipsoids drawn at the 50% probability level. Suffix A in the atom labels indicates symmetry (i) –x+1, –y+2, –z+1.

## supplementary crystallographic information

### Comment

Quinoline derivatives represent a major class of heterocycles, and a number of preparations have been known since the late 1800s (Kouznetsov *et al.*, 2005). The quinoline ring system occurs in various natural products, especially in alkaloids (Kouznetsov *et al.*, 2005). Some 3-substituted quinoline derivatives can be used as Platelet-derived growth factor (PDGF) receptors (Maguire *et al.*, 1994). In the course of exploring new quinoline derivatives, we obtained the title compound (I), and the synthesis and structure are reported here.

### Experimental

The precursor MBQ (10-methoxybenzo[*h*]quinoline) was synthesized according to Dong *et al.* (2011). Under a nitrogen atmosphere and at -78°C, a solution of n-butyllithium (0.16 mol) in anhydrous n-hexane (100 ml) was added portionwise with stirring to a solution of 9-bromoanthracene(14.5 g, 56.3 mmol) in anhydrous tetrahydrofuran (100 ml), and stirring continued for 50 min to form 9-Lithiumanthracene. Then a solution of MBQ (7.85 g, 37.5 mmol) in tetrahydrofuran (75 ml) was added dropwise with stirring over 2 h and the mixture was stirred for another 3 h. The resulting orange reaction mixture was poured over 400 g crushed ice and neutralised with 6 *M* HCl solution, and then the organic solvents, n-hexane and tetrahydrofuran, were removed by evaporation to give the crude product of the title compound as a dark-red solid (11.1 g). The crude product was collected by filtration and then washed well with a hot ethanol-water mixture (1/1 *v*/*v*). Finally, recrystallization from acetone gave a pure sample of the title compound as yellow crystals (3.1 g; yield 40.0% based on MBQ). ^1^H NMR (300 MHz, CDCl_3_): δ 7.87-7.17 (m, 16H; benzo[h]quinoline and anthracen rings), 3.86, 3.81 (d, 3H; OCH_3_), 2.18 (s, 3H; [(CH_3_)_2_CO]_0.5_).

### Refinement

Three reflections (001, 010, -101) have been omitted as systematic errors. The site occupancy factor of atoms belonging to the solvent molecule was fixed to give a total occupancy of 0.5, consistent with the area of the acetone CH_3_ peaks in the ^1^H NMR spectrum. The anisotropic displacement parameters of the heavy atoms in the disordered solvent molecule have been restrained to approximate an isotropic behaviour by the use of the ISOR command in SHELXL97. All H atoms were placed in geometrically calculated positions and refined using a riding model with C—H = 0.93 Å and *U*_iso_(H) = 1.2 *U*_eq_(C).

### Crystal data

**Table:**

C_28_H_19_NO·0.5C_3_H_6_O	*Z* = 2
*M**_r_* = 414.48	*F*(000) = 436
Triclinic, *P*1	*D*_x_ = 1.267 Mg m^−^^3^
*a* = 9.198 (3) Å	Mo *K*α radiation, λ = 0.71073 Å
*b* = 10.690 (4) Å	Cell parameters from 1500 reflections
*c* = 11.130 (4) Å	θ = 2.2–26.5°
α = 95.224 (4)°	µ = 0.08 mm^−^^1^
β = 91.484 (5)°	*T* = 293 K
γ = 94.064 (5)°	Block, yellow
*V* = 1086.6 (6) Å^3^	0.30 × 0.20 × 0.20 mm

### Data collection

**Table:**

Bruker SMART APEX CCD diffractometer	3688 independent reflections
Radiation source: fine-focus sealed tube	2474 reflections with *I* > 2σ(*I*)
Graphite monochromator	*R*_int_ = 0.011
φ and ω scan	θ_max_ = 25.0°, θ_min_ = 2.2°
Absorption correction: multi-scan (*SADABS*; Sheldrick, 1996)	*h* = −7→10
*T*_min_ = 0.977, *T*_max_ = 0.985	*k* = −12→12
4167 measured reflections	*l* = −13→11

### Refinement

**Table:**

Refinement on *F*^2^	Primary atom site location: structure-invariant direct methods
Least-squares matrix: full	Secondary atom site location: difference Fourier map
*R*[*F*^2^ > 2σ(*F*^2^)] = 0.069	Hydrogen site location: inferred from neighbouring sites
*wR*(*F*^2^) = 0.160	H-atom parameters constrained
*S* = 1.01	*w* = 1/[σ^2^(*F*_o_^2^) + (0.0496*P*)^2^ + 0.6424*P*] where *P* = (*F*_o_^2^ + 2*F*_c_^2^)/3
3688 reflections	(Δ/σ)_max_ < 0.001
300 parameters	Δρ_max_ = 0.58 e Å^−^^3^
24 restraints	Δρ_min_ = −0.36 e Å^−^^3^

### Special details

**Table:**

Geometry. All esds (except the esd in the dihedral angle between two l.s. planes) are estimated using the full covariance matrix. The cell esds are taken into account individually in the estimation of esds in distances, angles and torsion angles; correlations between esds in cell parameters are only used when they are defined by crystal symmetry. An approximate (isotropic) treatment of cell esds is used for estimating esds involving l.s. planes.
Refinement. Refinement of F^2^ against ALL reflections. The weighted R-factor wR and goodness of fit S are based on F^2^, conventional R-factors R are based on F, with F set to zero for negative F^2^. The threshold expression of F^2^ > 2sigma(F^2^) is used only for calculating R-factors(gt) etc. and is not relevant to the choice of reflections for refinement. R-factors based on F^2^ are statistically about twice as large as those based on F, and R- factors based on ALL data will be even larger.

### Fractional atomic coordinates and isotropic or equivalent isotropic displacement parameters (Å^2^)

**Table:**

	*x*	*y*	*z*	*U*_iso_*/*U*_eq_	Occ. (<1)
N1	0.9385 (2)	0.7649 (2)	0.19927 (19)	0.0487 (6)	
O1	0.8308 (2)	0.55815 (19)	0.06758 (18)	0.0685 (6)	
C1	1.0227 (3)	0.8273 (3)	0.2862 (2)	0.0523 (7)	
C2	1.0377 (4)	0.9587 (3)	0.3040 (3)	0.0806 (11)	
H2	1.0988	0.9996	0.3654	0.097*	
C3	0.9605 (4)	1.0255 (3)	0.2293 (4)	0.0914 (12)	
H3	0.9682	1.1131	0.2401	0.110*	
C4	0.7849 (4)	1.0318 (3)	0.0594 (4)	0.0956 (12)	
H4	0.7913	1.1194	0.0684	0.115*	
C5	0.6963 (4)	0.9694 (4)	−0.0260 (4)	0.0922 (12)	
H5	0.6413	1.0152	−0.0751	0.111*	
C6	0.5836 (4)	0.7764 (4)	−0.1350 (3)	0.0862 (11)	
H6	0.5299	0.8249	−0.1825	0.103*	
C7	0.5664 (4)	0.6499 (5)	−0.1524 (3)	0.0912 (12)	
H7	0.4987	0.6116	−0.2103	0.109*	
C8	0.6485 (3)	0.5765 (4)	−0.0852 (3)	0.0751 (9)	
H8	0.6364	0.4893	−0.0996	0.090*	
C9	0.7480 (3)	0.6299 (3)	0.0028 (2)	0.0581 (7)	
C10	0.8701 (3)	0.9638 (3)	0.1371 (3)	0.0717 (9)	
C11	0.8621 (3)	0.8311 (3)	0.1236 (2)	0.0528 (7)	
C12	0.6825 (3)	0.8358 (3)	−0.0450 (3)	0.0699 (9)	
C13	0.7672 (3)	0.7633 (3)	0.0278 (2)	0.0555 (7)	
C14	1.1068 (3)	0.7524 (2)	0.3671 (2)	0.0486 (7)	
C15	1.0442 (3)	0.7100 (2)	0.4715 (2)	0.0510 (7)	
C16	0.8982 (3)	0.7313 (3)	0.5034 (3)	0.0609 (8)	
H16	0.8414	0.7761	0.4546	0.073*	
C17	0.8410 (4)	0.6874 (3)	0.6035 (3)	0.0732 (9)	
H17	0.7460	0.7034	0.6232	0.088*	
C18	0.9233 (4)	0.6178 (3)	0.6782 (3)	0.0770 (9)	
H18	0.8818	0.5871	0.7460	0.092*	
C19	1.0615 (4)	0.5955 (3)	0.6525 (3)	0.0698 (9)	
H19	1.1141	0.5486	0.7024	0.084*	
C20	1.1291 (3)	0.6424 (3)	0.5499 (2)	0.0551 (7)	
C21	1.2725 (3)	0.6238 (3)	0.5236 (3)	0.0606 (8)	
H21	1.3280	0.5823	0.5763	0.073*	
C22	1.3367 (3)	0.6650 (2)	0.4208 (3)	0.0535 (7)	
C23	1.4854 (3)	0.6474 (3)	0.3929 (3)	0.0662 (8)	
H23	1.5430	0.6078	0.4456	0.079*	
C24	1.5438 (3)	0.6870 (3)	0.2919 (3)	0.0734 (9)	
H24	1.6402	0.6736	0.2750	0.088*	
C25	1.4589 (3)	0.7485 (3)	0.2122 (3)	0.0711 (9)	
H25	1.4998	0.7750	0.1424	0.085*	
C26	1.3194 (3)	0.7698 (3)	0.2352 (3)	0.0605 (8)	
H26	1.2662	0.8117	0.1814	0.073*	
C27	1.2511 (3)	0.7294 (2)	0.3405 (2)	0.0500 (7)	
C28	0.8286 (4)	0.4265 (3)	0.0294 (3)	0.0799 (10)	
H28A	0.7327	0.3877	0.0388	0.120*	
H28B	0.8978	0.3882	0.0778	0.120*	
H28C	0.8538	0.4154	−0.0538	0.120*	
O2	0.6026 (8)	0.9238 (7)	0.5926 (7)	0.144 (2)	0.50
C29	0.529 (2)	0.9719 (13)	0.5379 (16)	0.165 (3)	0.50
C30	0.3978 (8)	0.9798 (6)	0.5752 (6)	0.173 (3)	
H30A	0.3522	0.8966	0.5787	0.260*	
H30B	0.3429	1.0240	0.5203	0.260*	
H30C	0.4007	1.0245	0.6541	0.260*	

### Atomic displacement parameters (Å^2^)

**Table:**

	*U*^11^	*U*^22^	*U*^33^	*U*^12^	*U*^13^	*U*^23^
N1	0.0432 (12)	0.0504 (13)	0.0536 (14)	0.0073 (10)	−0.0003 (10)	0.0094 (11)
O1	0.0708 (14)	0.0658 (14)	0.0672 (13)	0.0075 (10)	−0.0151 (11)	−0.0006 (11)
C1	0.0477 (16)	0.0492 (17)	0.0597 (18)	0.0056 (13)	−0.0055 (13)	0.0038 (13)
C2	0.084 (2)	0.0502 (19)	0.104 (3)	0.0075 (17)	−0.033 (2)	−0.0007 (18)
C3	0.098 (3)	0.0456 (19)	0.130 (3)	0.0118 (18)	−0.027 (2)	0.012 (2)
C4	0.089 (3)	0.070 (2)	0.133 (4)	0.012 (2)	−0.020 (3)	0.044 (2)
C5	0.073 (2)	0.102 (3)	0.111 (3)	0.013 (2)	−0.017 (2)	0.059 (3)
C6	0.065 (2)	0.133 (4)	0.065 (2)	0.013 (2)	−0.0119 (17)	0.034 (2)
C7	0.071 (2)	0.133 (4)	0.069 (2)	0.008 (2)	−0.0160 (18)	0.009 (2)
C8	0.062 (2)	0.098 (3)	0.063 (2)	0.0026 (18)	−0.0064 (16)	−0.0007 (18)
C9	0.0472 (17)	0.079 (2)	0.0489 (17)	0.0050 (15)	0.0015 (13)	0.0065 (15)
C10	0.066 (2)	0.061 (2)	0.091 (2)	0.0084 (16)	−0.0095 (18)	0.0239 (18)
C11	0.0435 (15)	0.0582 (18)	0.0600 (17)	0.0074 (13)	0.0043 (13)	0.0191 (14)
C12	0.0557 (19)	0.094 (3)	0.066 (2)	0.0079 (17)	0.0020 (15)	0.0356 (18)
C13	0.0417 (15)	0.076 (2)	0.0515 (17)	0.0061 (14)	0.0047 (12)	0.0193 (15)
C14	0.0499 (16)	0.0417 (15)	0.0531 (16)	0.0053 (12)	−0.0082 (13)	−0.0011 (12)
C15	0.0535 (17)	0.0431 (15)	0.0547 (17)	0.0050 (12)	−0.0056 (13)	−0.0032 (13)
C16	0.0544 (18)	0.0600 (19)	0.068 (2)	0.0064 (14)	−0.0024 (15)	0.0010 (15)
C17	0.063 (2)	0.078 (2)	0.077 (2)	0.0023 (17)	0.0109 (17)	0.0006 (18)
C18	0.087 (3)	0.081 (2)	0.062 (2)	−0.001 (2)	0.0133 (18)	0.0083 (18)
C19	0.084 (2)	0.071 (2)	0.0567 (19)	0.0106 (17)	−0.0004 (17)	0.0108 (16)
C20	0.0614 (19)	0.0514 (17)	0.0523 (17)	0.0072 (14)	−0.0043 (14)	0.0022 (13)
C21	0.0648 (19)	0.0574 (18)	0.0607 (19)	0.0163 (14)	−0.0147 (15)	0.0072 (14)
C22	0.0546 (17)	0.0458 (16)	0.0593 (18)	0.0085 (13)	−0.0083 (14)	−0.0002 (13)
C23	0.0550 (19)	0.0616 (19)	0.082 (2)	0.0140 (15)	−0.0098 (16)	0.0014 (17)
C24	0.0534 (19)	0.074 (2)	0.093 (3)	0.0103 (16)	0.0082 (18)	0.0024 (19)
C25	0.064 (2)	0.075 (2)	0.077 (2)	0.0077 (17)	0.0115 (17)	0.0099 (17)
C26	0.0601 (19)	0.0588 (18)	0.0633 (19)	0.0075 (14)	−0.0011 (15)	0.0071 (15)
C27	0.0516 (16)	0.0428 (15)	0.0550 (17)	0.0053 (12)	−0.0041 (13)	0.0006 (13)
C28	0.086 (2)	0.068 (2)	0.083 (2)	0.0024 (18)	−0.0104 (19)	−0.0057 (18)
O2	0.139 (5)	0.144 (5)	0.149 (5)	0.022 (4)	−0.012 (4)	0.018 (4)
C29	0.202 (5)	0.118 (4)	0.164 (5)	−0.002 (4)	−0.041 (5)	−0.026 (4)
C30	0.213 (5)	0.123 (3)	0.171 (5)	−0.003 (4)	−0.044 (4)	−0.027 (3)

### Geometric parameters (Å, º)

**Table:**

N1—C1	1.323 (3)	C16—C17	1.352 (4)
N1—C11	1.362 (3)	C16—H16	0.9300
O1—C9	1.358 (3)	C17—C18	1.406 (4)
O1—C28	1.431 (3)	C17—H17	0.9300
C1—C2	1.397 (4)	C18—C19	1.343 (4)
C1—C14	1.495 (3)	C18—H18	0.9300
C2—C3	1.362 (4)	C19—C20	1.428 (4)
C2—H2	0.9300	C19—H19	0.9300
C3—C10	1.391 (5)	C20—C21	1.383 (4)
C3—H3	0.9300	C21—C22	1.393 (4)
C4—C5	1.334 (5)	C21—H21	0.9300
C4—C10	1.433 (4)	C22—C27	1.431 (3)
C4—H4	0.9300	C22—C23	1.432 (4)
C5—C12	1.420 (5)	C23—C24	1.348 (4)
C5—H5	0.9300	C23—H23	0.9300
C6—C7	1.346 (5)	C24—C25	1.404 (4)
C6—C12	1.411 (5)	C24—H24	0.9300
C6—H6	0.9300	C25—C26	1.346 (4)
C7—C8	1.381 (5)	C25—H25	0.9300
C7—H7	0.9300	C26—C27	1.430 (4)
C8—C9	1.379 (4)	C26—H26	0.9300
C8—H8	0.9300	C28—H28A	0.9600
C9—C13	1.425 (4)	C28—H28B	0.9600
C10—C11	1.409 (4)	C28—H28C	0.9600
C11—C13	1.464 (4)	O2—C29	1.083 (13)
C12—C13	1.425 (4)	C29—C29^i^	1.22 (3)
C14—C27	1.401 (4)	C29—C30	1.30 (2)
C14—C15	1.407 (4)	C30—H30A	0.9600
C15—C16	1.426 (4)	C30—H30B	0.9600
C15—C20	1.433 (4)	C30—H30C	0.9600
			
C1—N1—C11	118.9 (2)	C15—C16—H16	119.5
C9—O1—C28	117.7 (2)	C16—C17—C18	120.9 (3)
N1—C1—C2	123.1 (3)	C16—C17—H17	119.6
N1—C1—C14	117.8 (2)	C18—C17—H17	119.6
C2—C1—C14	119.1 (2)	C19—C18—C17	120.5 (3)
C3—C2—C1	118.4 (3)	C19—C18—H18	119.8
C3—C2—H2	120.8	C17—C18—H18	119.8
C1—C2—H2	120.8	C18—C19—C20	121.2 (3)
C2—C3—C10	120.5 (3)	C18—C19—H19	119.4
C2—C3—H3	119.8	C20—C19—H19	119.4
C10—C3—H3	119.8	C21—C20—C19	122.2 (3)
C5—C4—C10	119.9 (3)	C21—C20—C15	119.3 (3)
C5—C4—H4	120.0	C19—C20—C15	118.4 (3)
C10—C4—H4	120.0	C20—C21—C22	122.3 (3)
C4—C5—C12	122.7 (3)	C20—C21—H21	118.9
C4—C5—H5	118.6	C22—C21—H21	118.9
C12—C5—H5	118.6	C21—C22—C27	118.6 (3)
C7—C6—C12	120.4 (3)	C21—C22—C23	122.9 (3)
C7—C6—H6	119.8	C27—C22—C23	118.5 (3)
C12—C6—H6	119.8	C24—C23—C22	121.5 (3)
C6—C7—C8	120.6 (3)	C24—C23—H23	119.3
C6—C7—H7	119.7	C22—C23—H23	119.3
C8—C7—H7	119.7	C23—C24—C25	120.0 (3)
C9—C8—C7	121.3 (4)	C23—C24—H24	120.0
C9—C8—H8	119.3	C25—C24—H24	120.0
C7—C8—H8	119.3	C26—C25—C24	121.0 (3)
O1—C9—C8	121.5 (3)	C26—C25—H25	119.5
O1—C9—C13	118.0 (2)	C24—C25—H25	119.5
C8—C9—C13	120.5 (3)	C25—C26—C27	121.6 (3)
C3—C10—C11	118.0 (3)	C25—C26—H26	119.2
C3—C10—C4	121.5 (3)	C27—C26—H26	119.2
C11—C10—C4	120.5 (3)	C14—C27—C26	122.5 (2)
N1—C11—C10	121.2 (3)	C14—C27—C22	120.1 (3)
N1—C11—C13	119.5 (3)	C26—C27—C22	117.4 (3)
C10—C11—C13	119.3 (2)	O1—C28—H28A	109.5
C6—C12—C5	119.5 (3)	O1—C28—H28B	109.5
C6—C12—C13	120.7 (3)	H28A—C28—H28B	109.5
C5—C12—C13	119.8 (3)	O1—C28—H28C	109.5
C12—C13—C9	116.5 (3)	H28A—C28—H28C	109.5
C12—C13—C11	117.7 (3)	H28B—C28—H28C	109.5
C9—C13—C11	125.7 (2)	C29^i^—C29—O2	167 (4)
C27—C14—C15	120.3 (2)	C29^i^—C29—C30	76.3 (19)
C27—C14—C1	119.1 (2)	O2—C29—C30	117 (2)
C15—C14—C1	120.5 (2)	C29—C30—H30A	109.5
C14—C15—C16	122.7 (2)	C29—C30—H30B	109.5
C14—C15—C20	119.4 (2)	H30A—C30—H30B	109.5
C16—C15—C20	117.9 (3)	C29—C30—H30C	109.5
C17—C16—C15	121.0 (3)	H30A—C30—H30C	109.5
C17—C16—H16	119.5	H30B—C30—H30C	109.5

Symmetry code: (i) −*x*+1, −*y*+2, −*z*+1.
